# Lateral Extra-articular Tenodesis With Anterior Cruciate Ligament Reconstruction in Pediatric and Skeletally Immature Patients: A Systematic Review and Meta-analysis

**DOI:** 10.1177/03635465251407326

**Published:** 2026-01-21

**Authors:** Satyavenkata Kotipalli, Thomas Haidl, Prushoth Vivekanantha, Darren de SA, Jeffrey Kay

**Affiliations:** * Michael DeGroote School of Medicine, McMaster University, Hamilton, Ontario, Canada; † Division of Orthopaedic Surgery, Department of Surgery, McMaster University, Hamilton, Ontario, Canada; Investigation performed at the Division of Orthopedic Surgery, McMaster University, Hamilton, Ontario, Canada

**Keywords:** anterior cruciate ligament reconstruction, iliotibial band, lateral extra-articular tenodesis, return to sport, pediatric

## Abstract

**Background:**

Lateral extra-articular tenodesis (LET), alongside anterior cruciate ligament reconstruction (ACLR), has been shown to improve rerupture and rotational laxity in patients <25 years. However, safety and efficacy in both general pediatric (<18 years) and skeletally immature patients are important to identify.

**Purpose:**

To assess clinical outcomes and complications after the LET procedure with ACLR in the pediatric and skeletally immature population.

**Study Design:**

Meta-analysis; Level of evidence, 4.

**Methods:**

Three databases were searched on December 5, 2024. Data were collected on study characteristics, demographics, surgical details, LET indications, patient-reported outcome measures, return to sport (RTS), rerupture rates, and complications. A meta-analysis of graft rerupture and RTS was performed using a Mantel-Haenszel and fixed-effects model (pooled effect measure: odds ratio [OR] with 95% CI).

**Results:**

Nine studies comprising 317 patients (318 knees) were included, of whom 204 patients (205 knees) were skeletally immature. The mean age of all patients and skeletally immature patients was 14.6 years (range, 8-18 years) and 13.6 years (range, 8-16.1 years), respectively. Common indications for LET included a grade 2+ pivot shift and intention to return to a high level of sport. The pooled RTS rate of ACLR+LET was 96% (92%-99%; *I*^*2*^ = 48%) and 98% (94%-100%; *I*^*2*^ = 39%) in general pediatric and skeletally immature patients, respectively. The rerupture rate after ACLR+LET was 1.6% and 2.4% in general pediatric and skeletally immature patients, respectively. Pooled data consisting of 119 patients who underwent ACLR+LET and 87 patients with isolated ACLR found ACLR+LET to have a significantly lower rate of ACLR graft reruptures compared with isolated ACLR of 0.8% and 12.6%, respectively (*I*^*2*^ = 0%; OR = 0.12; 95% CI, 0.03-0.53; *P* = .0036). ACLR+LET was also found to have a significantly higher RTS rate compared with isolated ACLR (92.4% vs 80.5%, respectively) (*I*^*2*^ = 0%; OR, 3.06; 95% CI, 1.3-7.18; *P* = .0104). There were 2 reports of growth disturbances (0.63%), with 1 case being corrected by surgical epiphysiodesis and another being clinically asymptomatic.

**Conclusion:**

The LET procedure, as an adjunct to ACLR in pediatric and adolescent patients, has been shown to be safe with low complication rates—including physeal disturbance. Pooled data from the literature to date demonstrate that ACLR combined with an LET has a lower rate of graft rerupture while maintaining previously described high rates of RTS in pediatric patients compared with isolated ACLR.

Despite generally favorable outcomes after anterior cruciate ligament reconstruction (ACLR), younger patients remain at a higher risk of graft failure and revision surgery.^12,23^ Recent systematic reviews and meta-analyses found rerupture rates just >10% in these younger populations, a failure rate still far higher than their adult counterparts at closer^2,6,23,24,46^ to 4%. There are a variety of factors that may influence the rerupture rate—including preoperative high-grade pivot shift,^18,30^ high posterior tibial slope,^27^ and different graft types.^5,6,38,40,42,56^ Reliable methods to improve graft integrity and knee stability in young populations remain of great interest and importance.^27^

To maintain rotatory stability, the anterolateral complex (ALC) is understood to work synergistically with the ACL.^29,35,36,54^ With growing recognition of the importance of the ALC in stability and unsatisfactory rotary control from modified intra-articular techniques alone in high-risk young athletes, lateral extra-articular procedures (LEAPs) are increasingly relevant in ACLR.^14^ Examples of LEAPs include the modified Lemaire, the Arnold-Coker modification of the MacIntosh Technique, anterolateral ligament (ALL) reconstruction, the *over-the-top* Marcacci-Zaffagnini, and the Kocher-Micheli.^50^ Both the modified Lemaire and the Arnold-Coker modification of the MacIntosh technique are representative of a lateral extra-articular tenodesis (LET)—a nonanatomic soft tissue restraint on the lateral compartment of the knee providing mechanical stabilization.^14,50^

In recent years, concomitant LEAP procedures have demonstrated favorable outcomes in both adult and young adult populations, reducing rates of graft failure, improving patient outcome measures, and reducing anterolateral rotary instability as measured by the pivot shift test.^11,14,19,51^ Recognizing this utility of the LET in conjunction with ACLR and the high rates of graft failure among skeletally immature and pediatric populations, there is high interest in the efficacy and safety of LET procedures in pediatric and adolescent populations, particularly in the skeletally immature population, where there can be a risk of physeal disturbance. Given the demonstrated benefit of LET on graft failure and patient outcomes in adult populations, most notably shown by the investigators of the STABILITY I trial for high-risk individuals undergoing ACLR with HT autografts,^14^ it is important to further investigate the safety and utility of LET procedures in pediatric and adolescent populations. The purpose of this systematic review is to assess the utility, safety, and outcomes of the LET procedure and ACLR in pediatric and adolescent populations, with particular attention to skeletally immature individuals. Specifically, the study will compare the rates of rerupture and RTS in pediatric patients with isolated ACLR and ACLR+LET and assess the number of complications—including physeal disturbance. Given the results from previous studies in adult populations, it is hypothesized that the addition of the LET procedure in pediatric patients undergoing ACLR will result in lower rerupture rates with minimal physeal-related complications.

## Methods

This systematic review adheres to the updated PRISMA (Preferred Reporting Items for Systematic Reviews and Meta-Analyses)^37^ and the AMSTAR 2 (second Assessment of Multiple Systematic Reviews)^47^ guidelines for conducting and reporting systematic reviews.

### Search Strategy

Three online databases (PubMed, MEDLINE, EMBASE) were searched from inception to December 5, 2024, for studies reporting outcomes of LET, in addition to ACLR, in pediatric and adolescent populations on the COVIDENCE platform (Veritas Health Innovation). Search terms can be found in Appendix Table A1.

The inclusion criteria were as follows: (1) Studies with >5 patients undergoing any LET procedure in addition to an intra-articular ACLR; and (2) a mean age <18 years. The exclusion criteria were as follows: (1) ALL reconstruction; (2) any technique for which the LET and ACLR were a single contiguous graft as in isolated over-the-top ACLR and LET techniques; (3) systematic reviews; (4) meta-analyses; (5) review articles; (6) book chapters; (7) studies with <5 patients; and (8) technical note papers.

### Study Screening

All articles retrieved using the search terms were screened by 2 separate authors (S.K. and T.H.) based on the abstract and title. Conflicts of exclusion/inclusion were resolved once consensus was reached by both authors, or, in the case of continued discrepancy, by consulting a senior author (P.V.). Full-texts were then reviewed independently by the 2 authors, and conflicts were resolved once consensus was reached.

### Quality Assessment

Methodological quality of the screened studies was assessed using the Methodological Index for Non-Randomized Studies (MINORS) criteria.^49^ Per the MINORS criteria, the maximum cumulative score for a noncomparative study is 16, and the maximum score for a comparative study^49^ is 24. Per a priori designation, noncomparative studies were categorized as follows: 0-4 very low quality, 5-7 low quality, 8-12 fair quality, and ≥13 high quality.^4^ Comparative studies were categorized by the following: 0-6 very low quality, 7-10 low quality, 11-15 fair quality, 16-20 good quality, and ≥20 high quality.^4^

### Assessment of Agreement

Inter-reviewer agreement was assessed using the kappa (κ) statistic at all steps throughout the screening process. A priori classification criteria for the kappa statistic were employed as follows: 0.91-0.99, almost perfect agreement; 0.71-0.90, considerable agreement; 0.61-0.70, high agreement; 0.41-0.60, moderate agreement; 0.21-0.40, fair agreement; and a value ≤0.20 as no agreement.^4,33^

### Data Abstraction

Data were collected and summarized from screened articles by 2 independent authors (S.K. and T.H.) and compiled within a Google Sheets spreadsheet template generated before abstraction (Google LLC). Descriptive data—including patient age, sex, skeletal maturity status, and follow-up time—were recorded. Details regarding the ACLR and extra-articular operation were recorded—including indications, surgical details, postoperative rehabilitation, and concomitant injuries and procedures.

### Outcomes Assessed

Across studies included in this review, patient-reported outcome measures (PROMs) included the following: Knee injury and Osteoarthritis Outcome Score (KOOS)^45^; International Knee Documentation Committee (IKDC); Pediatric (Pedi-IKDC) subjective knee evaluation form scores^22,26^; Lysholm scores^3^; Tegner activity scale scores^3^; Single Assessment Numeric Evaluation scores^48^; and Pediatric Functional Activity Brief Scale (Pedi-FABS) score.^10^ Rates of graft reruptures in each of the groups were recorded along with measures of anterior/rotational instability. Additional collected patient outcomes included return to sport (RTS) rates and reported complications.

### Statistical Analysis

The extracted data were compiled for a statistical summary in a Google Sheets spreadsheet (Google LLC). Means summarizing grouped study data were calculated as weighted arithmetic means, while percentages were calculated as simple counts over the total relevant patient population.

Meta-analyses were performed using the DataParty platform. The statistical significance threshold was set a priori at *P* < .05. Pairwise dichotomous meta-analysis was performed using a Mantel-Haenszel^31^ and fixed effects model (given an anticipated low degree of statistical heterogeneity) for the dichotomous outcome variables of graft rerupture and RTS. Pooled effect estimates were derived by calculating the mean odds ratio (OR) for each dichotomous variable, along with its 95% CI. Forest plots were generated for graft rerupture and RTS, and the *I*^*2*^ test was used to assess heterogeneity. To estimate the pooled RTS rate, a meta-analysis of proportions was conducted. To establish the variance of the raw proportions, a Freeman-Tukey transformation was applied. The transformed proportions were then combined using the DerSimonian-Laird random effects model (to incorporate the anticipated heterogeneity).^7^ The proportions were back-transformed using an equation derived by Miller. *I*^*2*^ tests were used to assess for heterogeneity. “Low,”“moderate,” and “high” values are identified by *I*^*2*^ values of 25%-49%, 50%-74%, and >75% respectively.^20^

## Results

### Literature Search

**Figure 1.**
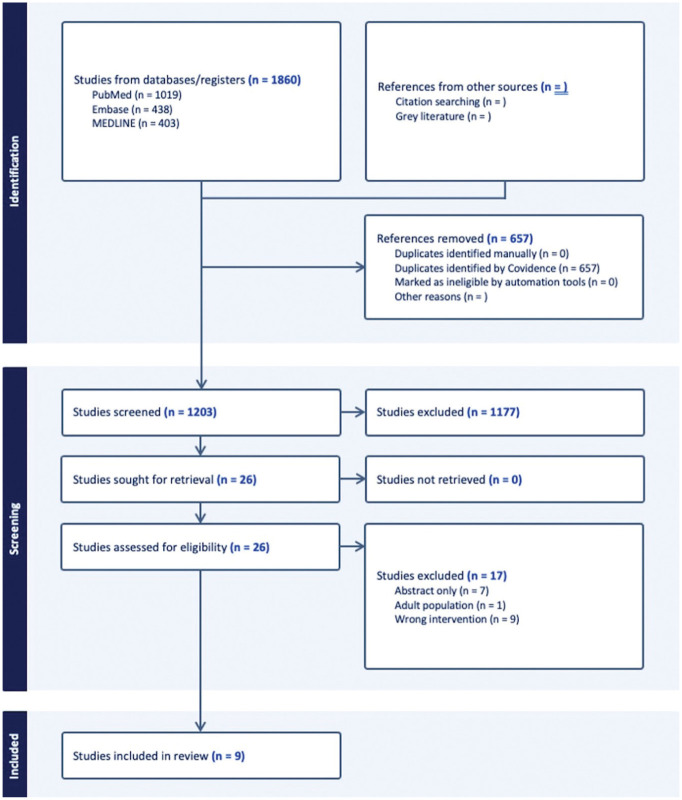
PRISMA flow diagram representing a systematic review of level 2, 3, and 4 studies on studies involving ACLR+LET in the youth population. ACLR, anterior cruciate ligament reconstruction; LET, lateral extra-articular tenodesis; PRISMA, Preferred Reporting Items for Systematic Reviews and Meta-analyses.

The initial search across PubMed, Embase, and MEDLINE yielded 1860 studies, with 657 identified as duplicates. Out of the remaining 1203 studies, 26 were selected for full-text review after title and abstract screening. Nine studies^8,13,15,16,17,34,39,43,55^ matched the eligibility criteria and were selected for final analysis (Figure 1). There was moderate agreement during title and abstract screening (*k* = 0.579; 95% CI, 0.441-0.718) and substantial agreement during full-text analysis (*k* = 0.752; 95% CI, 0.489-1).

### Study Quality

Of the 9 studies included in this systematic review, 5 were case series (level of evidence 4), and 4 were retrospective comparative studies (level of evidence 3). The mean MINORS score was 69.8% for the 5 case series and 79.2% for the 4 retrospective cohort studies (Table 1).

**Table 1:** Study and Patient Characteristics*^*a*^*

Author (Year)	Study Design, Level of Evidence	MINORS, %	No. of Patients With ACLR+LET, Knees	No. of Patients With Isolated ACLR	Age, Years, Mean (SD) [Range]	Women, n (%)	Time to Follow-up, months (SD) [Range]	Lost to Follow-Up (n, %)	Skeletally immature, n (%)
Ebert et al^8^ (2024)	Case series, 4	87.5	20	0	13.1 (1.8) [8-15]	7 (30)	Up to 24 months	0	20 (100)
Foissey et al^13^ (2022)	Case series, 4	68.8	20	0	14 (1.2) [10.9-16.1]	2 (10)	Minimum 42 months	0	20 (100)
Gomez- Caceres et al^15^ (2024)	Case series, 4	62.5	12	0	13.17 (0.9)	1 (8.33)	26 (12.6)	0	12 (100)
Green et al^16^ (2023)	Case series, 4	68.8	48	0	14.2 (1) [11-16]	21 (44)	40.8 (14.4) [24-84]	1 (2.04)	48 (100)
Guarino et al^17^ (2022)	Retrospective cohort, 3	62.5	42	0	17 (1.18) [14-18]	14 (33.3)	49.2 (31.2) [24-108]	0	NR
Monaco et al^34^ (2022)	Retrospective cohort, 3	75	71	40	16.1 (1.5) [13-17.6]	27 (38%)	47.9 (17.2) (24-89)	7 (5.93)	NR
Perelli et al^39^ (2022)	Prospective cohort, 2	75	32	34	13.8 (1.4) [12-16]	12 (37.5)	25.1 (2.2)	3 (4.35)	32 (100)
Retzky et al^43^ (2024)	Retrospective cohort, 3	87.5	16	13	13.82 (1.11)	10 (62.5)	36 (13.2)	0	16 (100)
Wilson et al^55^ (2019)	Case series, 4	68.8	56, 57	0	13 [11-16]	21 (36.8)	38.5 [24-78]	0	56 (100)

a ACLR, anterior cruciate ligament reconstruction; LET, lateral extra-articular tenodesis; MINORs, Methodological Index for Non-Randomized Studies; NR, not reported.

### Study Characteristics

Across the 9 studies included, there were 404 patients (405 knees), of whom 317 (318 knees) underwent ACLR with LET and 87 patients underwent isolated ACLR, serving as controls for comparative studies (Table 1). The weighted mean age of patients was 14.6 years (range, 8-18 years). Of the 317 patients, 94 (29.7%, range, 8.3%-62.5%) were reported as women. Seven studies,^15,16,17,34,39,43,55^ comprising 277 patients, reported a mean follow-up time of 40.7 months (range, 24-108 months), while 2 other studies^8,13^ reported a maximum follow-up time of 24 months and a minimum of 42 months, respectively. Of the 317 patients across 9 studies, 11 patients (3.5%; range, 0%-5.9%) were lost to follow-up. Of the 7 studies^8,13,15,16,39,43,55^ that reported skeletal immaturity, 204 patients (100%) were reported as skeletally immature, determined using bone age,^13,16,55^ open epiphyseal plates on magnetic resonance imaging,^8,39,43^ and open physeal plates by unspecified method^15^ (Table 1).

### Surgical Details

All 9 studies (318 knees) reported on the graft used for the LET. Seven studies (266 knees) used an iliotibial band (ITB) autograft,^8,15,16,17,34,39,43,55^ while 2 studies (52 knees) used a strip of fascia lata.^13,39^ The most common LET graft width was 1 cm reported in 6 studies with 199 knees^8,15,16,34,39,43^ and the most common length was 8 cm reported in 4 studies with 116 knees.^8,16,39,43^

Three different techniques were reported across 9 studies used for the LET. Six studies (148 knees) used a modified Lemaire technique,^8,13,15,17,39,43^ two studies (113 knees) used an Arnold-Coker modification of the MacIntosh procedure,^16,34^ and 1 study (57 knees) used an outside-in technique where the ITB was passed through and sutured at the femoral tunnel and fixed into the tibial tunnel used for the ACLR.^55^

Five studies (136 knees) using a modified Lemaire technique reported femoral fixation proximal and posterior to the femoral attachment of the LCL.^8,13,16,39,55^ The most common femoral fixation technique was suture anchors used in 3 studies (84 knees).^8,39,43^ Three studies (88 knees) reported femoral fixation distal to the distal femoral physis,^8,13,16^ while 3 studies (101 knees) reported femoral fixation proximal to the distal femoral physis.^15,39,55^ Four studies reporting on 108 knees used intraoperative imaging or fluoroscopy to avoid the physis.^15,16,39,43^

Four studies using the modified Lemaire technique (116 knees) reported fixation at 30° of knee flexion.^8,16,39,43^ Four studies (108 knees) reported neutral tibial rotation during fixation.^15,16,39,43^ Both studies using the Arnold-Coker modification of the MacIntosh procedure reported fixation in 90° of knee flexion with maximal tibial external rotation.^17,34^ Full details of the LET grafts and technique can be found in Appendix Table A2 (available in the online version of this article).

Six studies ^13,16,17,34,39,55^ (270 patients) reported that the most common indications for LET were a high-grade pivot shift (grades 2-3) reported in 3 studies^16,17,34^ and intention to return to high-level or contact sports also reported in 3 studies.^17,34,55^ Rehabilitation protocols are reported in Appendix Table A3 (available online).

### Isolated ACLR vs ACLR+LET

**Figure 2.**
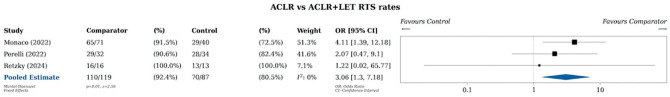
Forest plot showing pooled ORs comparing RTS rates in ACLR versus ACLR+LET. ACLR, anterior cruciate ligament reconstruction; LET, lateral extra-articular tenodesis; OR, odds ratio; RTS, return to sport.

**Figure 3.**
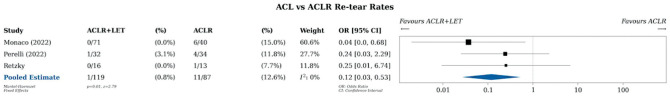
Forest plot demonstrating pooled ORs comparing rerupture rates in ACLR versus ACLR+LET. ACLR, anterior cruciate ligament reconstruction; LET, lateral extra-articular tenodesis; OR, odds ratio.

Three studies comparing ACLR with ACLR±LET reported on RTS and rerupture rates.^34,39,43^ These studies consisted of 87 patients who underwent only ACLR and 119 patients who underwent ACLR+LET. The RTS rate for patients with isolated ACLR was 80.5%, whereas it was 92.4% among patients who underwent ACLR+LET (*I*^*2*^ = 0%; OR, 3.06; 95% CI, 1.3-7.18; *P* = .0104) (Figure 2). Among the 87 patients who underwent isolated ACLR, there were 11 graft ruptures (12.6%), and there was 1 graft rupture (0.8%) among the 119 patients who underwent ACLR±LET (*I*^*2*^ = 0%; OR, 0.12, 95% CI, 0.03-0.53; *P* = .0036) (Figure 3).

### RTS and Complications

RTS rates were reported in 8 studies^8,15,16,17,34,39,43,55^ and RTS at preinjury level was reported in 3 studies^8,17,39^ (Table 2). The 8 studies reporting RTS included 297 patients, with a pooled RTS rate of 96% (92%-99%; *I*^*2*^ = 48%). Three studies (94 patients) reported that 79 (84%) returned to sport at a preinjury level. Two studies, with 32 and 56 patients, reported the time to RTS as 10.8 (SD, 1.4; range, 10.2-12.1) and 10.5 (range, 5.9-20.7) months, respectively^39,55^ (Table 2).

**Table 2:** RTS, Time to RTS, Rerupture, Time to Rerupture, and Revisions of Pediatric Patients Who Had Undergone ACLR With LET*^*a*^*

Author, Year	RTS, n, %	Time to RTS, Months, mean (SD) [Range]	Rerupture, n (%)	Time to Rerupture, Months, Mean (SD) [Range]	Revisions, n (%)
Ebert et al^8^ (2024)	RTS: 20/20 (100) Return to preinjury level: 18/20 (90)	24	0	NA	0
Foissey et al^13^ (2022)	NR	NR	1 (5)	32.9	1 (5)
Gomez-Caceres et al^15^ (2024)	12/12 (100)	NR	0	NA	0
Green et al^16^ (2023)	48/48 (100)	Within 2 years	0	NA	0
Guarino et al^17^ (2022)	RTS: 37/42 (88) RTS at preinjury level: 32/47 (76)	NR	0	NA	0
Monaco et al^34^ (2022)	65/71 (91.5)	NR	0	NR	0
Perelli et al^39^ (2022)	29/32 (90.6)	10.8 (1.4) [10.2-12.1]	1 (3.1)	NR	NR
Retzky et al^43^ (2024)	16/16 (100)	Reported at 2 years	0	NA	0
Wilson et al^55^ (2019)	52/56 (91)	10.5 [5.9-20.7]	3 (5.3)	24.7 [10-46]	NR

a NA, not applicable; NR, not reported; RTS, return to sport.

All 9 studies reported rerupture rates for ACLR (Table 2). Of 318 knees, there were 5 reruptures (1.6%) after surgery, all involving hamstring autografts. Two studies, each with 20 and 56 patients, reported mean times to rerupture of 32.9 and 24.7 months (range, 10-46 months), respectively.^13,55^

Complications varied widely between studies, with full details found in Table 3. Of the 318 knees that underwent ACLR+LET, major complications included 11 (3.5%) meniscus-related complications, 3 infection-related complications (0.9%), and 2 reports of growth being affected (0.6%). One report of growth disorders involved the operated leg being longer than the nonoperated leg, requiring surgical epiphysiodesis of the nonoperated knee for correction 40 months postoperatively.^13^ Another study found a leg length discrepancy of 12 mm and perioperative grade 2 valgus; however, it did not require correction as there was no symptomatic deformity.^55^ In total, 2 studies (89 knees) found 4 patients (1.26%) with an increased valgus grade on the operated knee compared with the contralateral side.^39,55^ One study in which 2 patients underwent simultaneous implant-mediated guided growth procedures for genu valgum also reported 2 hardware removal procedures^16^ (Table 3). Two studies reported on the flexion and extension range of motion in postoperative patients who underwent ACLR+LET. One study (20 patients) found the operated knee to have 3.4° less flexion and 0.3° less extension at final follow-up of 24 months compared with the contralateral knee, while the other study, with 71 patients, found a loss of 4.9° of flexion and 1.3° of extension at final follow-up of 47.9 months.^8,34^

**Table 3:** Complications of Pediatric Patients Who Have Undergone ACLR+LET*^*a*^*

Author (Year)	Complications, n (%)
Ebert et al^8^ (2024)	None
Foissey et al^13^ (2022)	ACL graft rupture: 1 (2.6) Meniscal suture failure: 1 (5) Anterior arthrofibrosis: 1 (5) Growth disorders: 1 (5)
Gomez-Caceres et al^15^ (2024)	None
Green et al^16^ (2023)	Meniscus-related procedures: 4 (All-epiphyseal: 1, transphyseal: 3) Quad donor site scar revision: 4 (All-epiphyseal: 0, transphyseal: 4) Irrigation and debridement: 2 (All-epiphyseal: 1, transphyseal: 1) Removal of hardware: 2 (All-epiphyseal: 1, transphyseal: 1)
Guarino et al^17^ (2022)	NR
Monaco et al^34^ (2022)	Secondary meniscal procedure: 5 (7) Excision of cyclops lesion: 1 (1.4) Manipulation under anesthesia: 1 (1.4) Arthroscopic lavage for septic arthritis: 1 (1.4) Anterior knee pain: 4 (5.6) Symptomatic tibial tunnel cyst: 1 (1.4) Dysesthesia: 3 (4.2) Hemarthrosis: 1 (1.4)
Perelli et al^39^ (2022)	Hematoma: 2 Arthrolysis for flexion/extension deficit: 1 Increased valgus in the operated leg on radiograph: 1
Retzky et al^43^ (2024)	Contralateral ACL tear: 1 Meniscal tear: 1 Suture granuloma: 4 Symptoms: 1 Reoperation: 5
Wilson et al^55^ (2019)	Leg length discrepancy: 1 Positive K–L in the lateral compartment: 3 Increased valgus grade compared with non-operated knee: 3

a ACL, anterior cruciate ligament; ACLR, ACL reconstruction; K–L, Kellgren–Lawrence; LET, lateral extra-articular tenodesis; NR, not reported.

### Patient-Reported Outcome Measures

Seven studies (281 patients) reported PROMs using either the IKDC or the Pedi-IKDC.^8,15,16,17,34,39,55^ Four studies^8,16,39,55^ consisting of 156 patients reported a weighted mean postoperative Pedi-IKDC of 91.24 (range, 89.13-97.6), while 3 studies,^15,17,34^ with 165 patients, reported a weighted mean postoperative IKDC score of 89.29 (range, 87.3-93.29). Two studies, with 12 and 42 patients, reported a Lysholm score of 95.08 (SD, 13.2) and 92 (SD, 9.6), respectively.^15,17^ Two studies, with 20 and 71 patients each, reported an overall KOOS score of 79 (SD, 2.9) and 90.5 (SD, 8.1), respectively.^8,34^ Two studies, with 42 and 71 patients each, reported postoperative Tegner scores of 7.8 (range, 6-10) and 7, respectively.^34,39^ Three studies^16,39,55^ (136 patients) reported a weighted mean postoperative Pedi-FABS score of 21.7. Full details can be found in Appendix Table A4 (available online).

### Skeletally Immature

Seven studies, with 204 patients (205 knees), reported their patient population as skeletally immature^8,13,15,16,39,43,55^ (Table 1). Of these 205 knees, 140 knees used a complete transphyseal technique, 13 knees used a partial epiphyseal technique, and 52 knees used an all-epiphyseal. Six studies,^8,15,16,39,43,55^ with 184 patients, reported an overall RTS rate, with a pooled RTS rate of 98% (94%-100%; *I*^*2*^ = 39%), with 2 studies^8,39^ ( 49 patients) reporting an overall RTS at a preinjury level rate of 95.9% (n = 47) (Table 2). There were 5 (2.4%) ACL rerupture cases among these 205 knees. Two studies reported mean times to rerupture of 32.9 and 24.7 months (range, 10-46 months), respectively.^13,55^ There were 2 (1%) recorded incidences of growth-related complications in skeletally immature patients across these 7 studies (Table 3).

## Discussion

The primary outcome of this study was that ACLR+LET is safe in the pediatric population with low rates of complications and physeal disturbances. Furthermore, ACLR+ LET has low rerupture rates in the pediatric population—including the skeletally immature subgroup, with rerupture rates of 1.6% and 2.4%, respectively. Furthermore, pooled data from 3 comparative studies found a significantly lower rerupture rate and a significantly higher RTS rate in the ACLR+LET group compared with the isolated ACLR group. In young athletes who plan to RTS at the preinjury level, the rate of secondary ACL injury has been reported^53^ as high as 23%.

The high risk of failure combined with the importance of maintaining athletic careers in this population underscores the significance of techniques/adjuvants that lower the rate of rerupture in the pediatric population. In this systematic review, lower rates of rerupture were observed in ACLR+LET compared with those reported in the current literature for isolated ACLR in the pediatric population. The proposed mechanism of the lower re-rupture rate is that the LET takes force off the ACL graft, allowing the new graft to heal.^9^

The most common PROMs reported across all studies were the IKDC and Pedi-IKDC, reported in 4 and 3 studies, respectively. The weighted mean postoperative IKDC score was 89.29, slightly higher than that reported in a recent systematic review, which found a postoperative IKDC score of 84.1 in pediatric patients who underwent isolated ACLR.^41^ This score is also consistent with the IKDC score of patients aged 18 to 25 years who underwent ACLR+LET in the STABILITY1 trials of 87.3 at the 2-year follow-up.^14^ Although further research should be done into the possible long-term complications of LET in pediatric patients, this suggests that, within the follow-up period reported in studies included in this systematic review, patients are generally very satisfied with their procedure.

In this review, a pooled RTS rate of 96% was found in ACLR+LET, with >80% able to return to their preinjury level of sport. These rates are consistent with pediatric isolated ACLR RTS rates reported in a 2018 systematic review and meta-analysis, which found a 92% RTS rate and an 81% return to competitive sports rate.^24,28^ The study above also found a graft rerupture rate of 13%, similar to the rerupture rate for isolated ACLR found in the present study's meta-analysis^24^ of 12.6%, but much higher than the overall rerupture rate for ACLR+LET of 1.6% found in this systematic review. This rate is more comparable with the rate of rerupture reported^14^ in high-risk adult patients in the STABILITY trial who had undergone ACLR+LET of 4%. Previous cadaveric studies^9,32^ have found that LET reduces the force on the ACL graft between 40% and 80%. Although the rerupture rate for ACLR+LET was found to be much lower than that of isolated ACLR, the sample size used in this meta-analysis was 119 patients, and larger prospective randomized controlled trials are needed to further support these findings. A high-grade pivot shift was a commonly reported indication for surgeons to add a LET procedure alongside ACLR in pediatric patients. As per the current literature, high-grade preoperative pivot shift has been found to increase postoperative pivot shift in isolated ACLR, and a lower postoperative pivot shift has been correlated with better functional outcomes and PROMs.^1,32,52^ The findings of this systematic review provide evidence that LET procedures should be strongly considered as an adjunct in pediatric patients undergoing ACLR, often with soft tissue grafts, especially those at higher risk of rerupture, such as high-level pivoting athletes and those with a high-grade pivot shift.

As highlighted previously, there have been concerns that adding a LET to ACLR in pediatric patients, particularly those who are skeletally immature, may increase the rate of growth disturbance and complications. Several technical approaches have been developed to avoid inducing further potential growth disturbances with a concomitant LET procedure. In techniques such as the modified Lemaire, femoral fixation of the ITB strip is done proximal and posterior to the origin of the lateral collateral ligament on the femur under fluoroscopic guidance, placing it superior to the open physis using anchors to avoid convergence with the ACLR tunnel,^8,16,21,43^ or by drilling a transosseous tunnel even more proximally.^39^ Other techniques avoid the physis by anchoring the LET all-epiphyseal using the ACLR tunnel,^13^ anchoring in an over-the-top position,^55^ or avoiding fixation altogether using the Arnold-Coker modification of the MacIntosh procedure, suturing the ITB graft back to its preserved insertion on the Gerdy tubercle.^17,34^ Recognizing the inherent risk of ACLR, growth disturbances were reported for only 2 studies in this review in the added presence of LET for 2 patients (0.63%) and required only 1 instance of correction.^13,55^ Low rates of growth disturbance support the notion that LET approaches do not increase the risk of growth disturbance in skeletally immature patients who underwent ACLR. Valgus deformity has also been reported as a potential complication of skeletally immature ACLR+LET and is proposed to result from lateral compartment constraint.^25^ Despite previous associations made between LET procedures, over constraint, and valgus angulation,^44^ greater operative limb valgus angulation was not observed in any of the patients in 7 of 9 studies. Where observed, the valgus interlimb difference was detectable radiographically with no reported functional alignment concerns or treatment required.^39,55^

Despite previous reports and theoretical concerns within the literature regarding LET, preliminary evidence in this review indicates that LET, in conjunction with ACLR, is safe in skeletally immature and pediatric populations.

A primary limitation of this review is that available studies were predominantly retrospective and limited to the levels 3 and 4 evidence. Given this limitation, our meta-analysis was performed using only level 3 comparative studies, which selection biases and outcome heterogeneity may theoretically limit; however, the calculated *I*^*2*^ value of zero for both RTS and ACLR rerupture rates indicates a low level of statistical heterogeneity in their findings. A further limitation arises from studies that use different surgical techniques, as the selection of a specific LET procedure is determined primarily by surgeon preference. This heterogeneity is further compounded by other surgical procedures used for intra-articular ACLR in the presence of LET. Further research at higher levels of evidence is warranted to validate the preliminary findings of this review.

## Conclusion

The LET procedure as an adjunct to ACLR in pediatric and adolescent patients is safe with low rates of complications—including physeal disturbance in skeletally immature individuals. Pooled data from the literature to date demonstrate that ACLR combined with an LET has a lower rate of graft rerupture while continuing to maintain previously described high rates of RTS in pediatric patients compared with isolated ACLR.

## Supplemental Material

sj-pdf-1-ajs-10.1177_03635465251407326 – Supplemental material for Lateral Extra-Articular Tenodesis With Anterior Cruciate Ligament Reconstruction in Pediatric and Skeletally Immature Patients: A Systematic Review and Meta-analysis Supplemental material, sj-pdf-1-ajs-10.1177_03635465251407326 for Lateral Extra-Articular Tenodesis With Anterior Cruciate Ligament Reconstruction in Pediatric and Skeletally Immature Patients: A Systematic Review and Meta-analysis by Satyavenkata Kotipalli, Thomas Haidl, Prushoth Vivekanantha, Darren de SA and Jeffrey Kay in The American Journal of Sports Medicine
