# Supplementary material for: Lateral Extra-articular Tenodesis With Anterior Cruciate Ligament Reconstruction in Pediatric and Skeletally Immature Patients: A Systematic Review and Meta-analysis
Source: Am J Sports Med. 2026 Jan 21;54(7):1747–56. doi: 10.1177/03635465251407326 (PMC13213001; doi:10.1177/03635465251407326)
Supplement: sj-pdf-1-ajs-10.1177_03635465251407326 – Supplemental material for Lateral Extra-Articular Tenodesis With Anterior Cruciate Ligament Reconstruction in Pediatric and Skeletally Immature Patients: A Systematic Review and Meta-analysis [file sj-pdf-1-ajs-10.1177_03635465251407326.pdf]

Lateral Extra-Articular Tenodesis with Anterior Cruciate Ligament Reconstruction in Pediatric and Skeletally Immature Patients is Safe and Yields Low Rates of Re-Rupture: A Systematic Review and Meta-Analysis

Appendix

Table A1: Search terms used for Pubmed, EMBASE and MEDLINE, including number of papers included.

Pubmed (n = 1019)	EMBASE (n = 438)	MEDLINE (n = 403)
Skeletally immature OR skeletal immaturity OR pediatric OR paediatric OR adolescent AND Lateral extra-articular OR tenodesis OR anterolateral ligament OR lemaire OR modified lemaire		

n = number of papers

Table A2: Surgical details of pediatric patients who have undergone ACLR alongside LET including ACL graft choice, technique, primary vs revision, LET indications, LET technique used, consideration for skeletally immature patients as well as any concomitant injuries and procedures.

**Author (Year)**	**Concomitant Injuries (n, list)**	**Concomitant procedures (n)**	**ACL graft**	**ACLR technique**	**Primary or revision (n)**	**LET graft details, fixation placement and**	**Brief LET technique description**	**Technique considerations for skeletally immature**	**Indications for LET**
Ebert (2024)	NR	Adjunct surgery: 8<br>Meniscectomy: 2<br>Meniscus Repair: 6	All soft tissue hamstring	Transphyseal	Primary: 20	1x8cm ITB<br>Fixed Posterior and proximal to LCL<br>Directed distally to avoid physis (Epiphyseal)<br>Bone anchor with sutures<br>Fixed at 30 degrees flexion	Modified Lemaire	Femoral bone anchor aimed distal to avoid distal femoral physis	NR
Foissey (2022)	NR	NR	All soft tissue hamstring	Partial Transphyseal	Primary: 20	Fascia Lata<br>Fixed posterior and proximal to match ALL<br>Physeal sparing femoral tunnel<br>Wires of ACL button used for fixation<br>Fixed in full extension	Modified Lemaire	NR	All patients under 25 undergo LET with ACLR at the institution in this study.

Gomez-Caceres (2024)	Lateral meniscus tear: 2<br>Medial meniscus tear: 3<br>Medial + lateral meniscus tear: 1	Meniscus repair: 4<br>Partial meniscectomy: 2	All soft tissue hamstring	Transphyseal	Primary: 12	1cm wide ITB<br>Fixed in same tunnel as ACL<br>Drilled proximal to distal femoral physis<br>Fixed with Interference screw, same as ACL<br>Fixed in full extension and neutral roation	Modified Lemaire	Avoided femoral physis when drilling tunnel under image intensifier guidance	NR
Green (2023)	NR	Implant-mediated guided growth for genu valgum: 2<br>Meniscal procedures: 4	All soft tissue quadriceps	All-epiphyseal: 10<br>transphyseal: 38	Primary: 46<br><br>Revision: 2	1x8cm from central third ITB<br>Fixed proximal and posterior to LCL and distal to femoral physis<br>Fixed with knotless suture anchor<br>Fixed in 30 degrees flexion and neutral rotation	Adjunctive modified Lemaire	If the patient was skeletally immature, all-epiphyseal technique was used.<br>If the patient had near closed physes, transphyseal technique was used.<br>Intraoperative imaging used.	If one or more of the following risk factors were present<br>- Grade 3 Pivot shift<br>- Hyperlaxity (Beighton score > 4)<br>- Knee recurvatum<br>- Revision ACLR<br>- Contralateral ACLR<br>- Chronic ACL Insufficiency
Guarino (2022)	NR	Medial meniscus repair: 10 (24%)<br>Lateral	All soft tissue hamstring: 34 (81%)<br>Tibialis	NR	Primary: 34<br>Revision: 8	Fixed with sutures back onto gerdys tubercle<br>Fixed in 90-100	Arnold-Coker modification of the MacIntosh	NR	Participation in contact sports, anterior cruciate ligament tear with pivot-shift test

		meniscus repair: 14 (33%)<br>Medial meniscectomy: 4 (10%)<br>Lateral meniscectomy: 3 (7%)	Tendon, allograft: 8 (19%)			knee flexion and external rotation	procedure		grade 2 or more, and knee hypermobility
Monaco (2022)	Lateral meniscal Tear: 8 (11.3%)<br>Medial meniscus tear: 9 (12.7%)<br>Lateral + medial meniscus tear: 2 (2.8%)<br>Chondral full-thickness lesion: 5 (7.0%)<br>Segond fracture: 3 (4.2%)	Lateral meniscus repair: 10<br>Medial meniscus repair: 10<br>Medial meniscectomy: 1<br>Lateral meniscectomy: 0<br>Microfracture: 5	All soft tissue hamstring	Transphyseal	Primary: 71	1x13xm at 3cm anterior to posterior border of ITB<br>Fixed in 90 degree flexion and maximal external tibial rotation<br>Fixed using absorbable periosteal stiches at level of gerdys tubercle	Arnold-Coker modification of the MacIntosh procedure	NR	If one or more of the following:<br>- pivot-shift grade 2 or 3<br>- high level of sporting activity defined as Tegner activity score ≥7<br>- participation in pivoting sports<br>- Segond fractures
Perelli (2022)	Meniscus tears: 22	Medial partial meniscectomy: 6<br>Meniscal	All soft tissue hamstring	Partial Transphyseal	Primary: 32	1x8cm middle third of fascia lata<br>Blind femoral tunnel, proximal to	Modified Lemaire	Femoral tunnel placed 1 cm proximal to suggested region	Routinely performed for all their pediatric patients

		repair: 18				femoral physis<br>Fixed proximal and posterior to LCL<br>Fixed with bioabsorbable interference screw<br>Fixed in 30 degrees flexion and neutral rotation		to avoid injury to physeal growth plate.<br>Fluoroscopy confirmed no growth plate injury.	
Retzky (2024)	NR	Medial meniscus repair: 6<br>Lateral meniscus repair: 10<br>Partial lateral meniscectomy: 1	All soft tissue quadriceps	All-epiphyseal:<br>Transphyseal: 13	Primary: 16	1x8cm central third of ITB<br>Secured proximal and posterior<br>Secure with suture anchor<br>Knee 30 degrees flexion with neutral tibial rotation<br>Secured back onto itself with suture, wrapping around LCL	Modified Lemaire	All-epiphyseal used fluoroscopy to ensure physis were not disrupted and to confirm hardware was outside of physis.	NR
Wilson (2019)	Lateral meniscus tear: 18<br>Medial meniscus tear: 8<br>Medial + lateral	Meniscus treatment: 26<br>Chondral Procedure: n = NR	All soft tissue hamstring	Transphyseal	Primary: 57	1.5cm x 13-15cm posterior ITB<br>Fixed proximal to femoral physis<br>Fixed with number 2 nonabsorbable suture<br>Fixed in extension and neutral rotation	Modified Lemaire with excess graft fixed into tibial tunnel in ACLR	NR	Skeletally immature patients with less than 3 years of growth remaining and intention to resume sports

	meniscus tear: 9								

ACL = Anterior cruciate ligament, ACLR = Anterior cruciate ligament reconstruction, LET = lateral extraarticular tenodesis, LCL = lateral collateral ligament, ITB = iliotibial band n = number of patients, NR = not reported.

Table A3: Description of rehabilitation protocol for pediatric patients who have undergone ACLR with LET.

Author (Year)	Rehabilitation
Ebert (2024)	Early bracing and touch weight bearing for the first 3-6 weeks.<br>Circulation, ROM, cycling, proprioception and strengthening from week 5-6.<br>End-range open chain exercises, running and high level rehab started at 4 months.<br>RTS permitted at 12 months after consultation.
Foissey (2022)	NR
Gomez-Caceres (2024)	Mobilization exercises of toes, ankle, knee and hip immediately post-op.<br>Flexion-extension exercises in early post-op period, but if meniscus was repaired avoid flexion beyond 90 degrees.<br>Isometric exercises for knee stabilizing muscles initiated day 1 post-op.<br>Partial weight bearing for 6 weeks<br>RTS permitted after 15 sessions of unrestricted training, at least 9 months post-op.
Green (2023)	No meniscus injury patients were placed in hinged knee brace locked in extension post-op and could weight bear with crutches as tolerated.<br>Brace was unlocked/discontinued after quadriceps muscle returned.<br>Standard ACL post-operative physical therapy protocol was followed.
Guarino (2022)	NR
Monaco (2022)	Knee extension brace for 4 weeks, all patients. Adjusted to allow 0-90 degrees after 2 weeks.<br>- No meniscal tear, weight bearing as tolerated from day 1 post-op.<br>- Meniscal tear, non weight bearing for 2 weeks.<br>Formal physical therapy on Day 2 post-op.<br>Prescribed muscle strengthening program 2 months post-op.

	Gradual sport-specific training and non-contact sport after 3 months.<br>Full return to sport allowed only after 9 months, ranging 9-12 months.
Perelli (2022)	No meniscal tear, full weight-bearing and full ROM from day 1.<br>Meniscal repair, weight bearing delayed until weeks 2-4.<br><br>Closed chain kinetic quadriceps strengthening exercises during the first 12 weeks.<br>Sport-specific training gradually after 6 months.<br>RTS between 10-12 months dependent on favourable assessment.
Retzky (2024)	Placed in a hinged knee brace and partial weight bearing for the first 4 weeks or 6 weeks if they had meniscus repair with ROM 0-90 degrees during this period.<br>After 4-6 weeks, ROM was increased and weight bearing as tolerated.<br>RTS was at minimum 9-12 months.
Wilson (2019)	Postoperative brace and cryotherapy followed by out-patient therapy.<br>Supervised Physical Therapy and 42/57 knees underwent RTP testing with Y-Balance and strength testing before RTS, at a minimum of 6 months post-op.

ACL = anterior cruciate ligament , ACLR = Anterior cruciate ligament reconstruction, LET = lateral extraarticular tenodesis, ROM = range of motion, NR = not reported, RTS = return to sport.

Table A4: Patient reported outcome measures of pediatric patients who have undergone ACLR with LET.

**Author (Year)**	**IKDC (Normal or pedi)**	**Lysholm**	**KOOS**	**Tegner**	**VAS**	**FABS**	**SANE**	**Tegner-Lysholm Knee Scores Scale**	**Signal Intensity Ratio**
Ebert (2024)	Pedi-IKDC<br>6 months postoperative: 81.4 (7.7)<br>12 months postoperative: 93.8 (7.2)<br>24 months postoperative: 97.6 (2.9)	NR	KOOS-ADL<br>6 months: 74.2 (3.6)<br>12 months: 78.2 (2.3)<br>24 months: 79 (2.9)	NR	NR	NR	NR	NR	NR
Foissey (2022)	NR	NR	NR	NR	NR	NR	NR	NR	NR
Gomez-Caceres (2024)	IKDC: 93.29 (11.04)	95.08 (13.2)	NR	9 (0.0)	NR	NR	NR	NR	NR
Green (2023)	Pedi-IKDC: 89.12 (12.74) [54.36-100]<br>HSS Pedi-FABS: 22.88	NR	NR	NR	NR	NR	93.19 (7.92) [70-100]	NR	NR

	(5.84) [11-30]								
Guarino (2022)	IKDC: 91.5 (8.0)	NR	NR	Preoperative: 8.1 [6 – 10] Postoperative: 7.8 [6 – 10]	NR	NR	NR	92.0 (9.6)	NR
Monaco (2022)	Subjective IKDC Score: 87.3 (9.8)<br><br>Pass: n=59 (83.1%)	NR	Overall KOOS: 90.5 (8.1)	7	NR	NR	NR	NR	NR
Perelli (2022)	Preoperative Pedi-IKDC: 53.9 (2.5) Postoperative Pedi-IKDC: 90.5 (9.6)	NR	NR	NR	NR	Preoperative Pedi-FABS: 19.2 (3.6) Postoperative Pedi-FABS: 18.5 (4.0)	NR	NR	NR
Retzky (2024)	NR	NR	NR	NR	NR	NR	NR	NR	6 months: 2.23 12 months: 2.03 24 months: 1.33
Wilson (2019)	Pedi-IKDC: 91.2 [46.8-100]	NR	NR	NR	NR	Pedi-FABS: 22.4 [4.0-30]	NR	NR	NR

ACLR = Anterior cruciate ligament reconstruction, LET = lateral extraarticular tenodesis, IKDC =

International Knee Documentation Committee, KOOS = Knee injury and osteoarthritis outcome score, VAS = Visual analog scale, FABS = Functional activity brief scale, SANE = Single assessment numeric evaluation, NR = not reported.
